# Caliban is a transcriptional target of p53 in response to DNA damage

**DOI:** 10.1371/journal.pone.0331141

**Published:** 2025-08-28

**Authors:** Jiaqian Cui, Haiyan Zhang, Yan Cheng, Xiaolin Bi, Dong Li

**Affiliations:** 1 Medical School of Nantong University, Nantong, China; 2 Institute of Cancer Stem Cell, Dalian Medical University, Dalian, China; Zhejiang Cancer Hospital, CHINA

## Abstract

Caliban, the *Drosophila* ortholog of human Nuclear export mediator factor (NEMF), is a recently identified regulator of the intrinsic apoptotic signaling pathway in response to DNA damage; however, the mechanism governing its expression after DNA damage remains unclear. In this study, we demonstrated that DNA damage upregulated *caliban* expression concomitant with p53 activation. Over-expression of *p53* upregulated the mRNA and protein levels of *caliban*. We characterized the core region of the *caliban* promoter, which exhibited enhanced activity following DNA damage or p53 activation. Further analysis of the *caliban* promoter revealed a p53-binding site that directly interacts with p53 in response to DNA damage. Moreover, mutation of this p53-binding site or knock-down of *p53* expression abolished the DNA damage-induced increase in *caliban* promoter activity, confirming p53’s critical role in regulating *caliban* expression. Taken together, our findings indicate that *caliban* is a direct transcriptional target of p53 in response to DNA damage.

## Introduction

The integrity of the genome is constantly challenged by various extrinsic DNA-damaging sources, such as ionizing, ultraviolet light (UV) radiation, and chemical agents in the environment [1,2]. To preserve genome integrity, cells have adapted a highly conserved and coordinated signaling networks, collectively termed as the DNA damage response (DDR), which determines cell fate following DNA damage through mechanisms like cell cycle arrest, DNA repair, or cell death [3,4]. The p53 tumor suppressor functions as a key node for orchestrating the DNA damage signaling via transcriptionally regulating multiple DDR downstream genes [5–8]. In response to DNA damage, the p53 protein is transiently stabilized and functionally activated via post-translational modifications. It then transcriptionally activates numerous genes by binding to its response elements within their regulatory regions [9–14]. These genes can promote cell survival by activating cell cycle checkpoints and DNA repair programs or induce cell death via triggering apoptosis [2]. Apoptosis induced by p53 activation serves as the final line of defense in eliminating the cells that contained excessive and irreparable damaged DNA. Over the past decades, numerous pro-apoptotic genes have been identified as transcriptional targets of p53 [15], such as *BAX* (Bcl-2-associated X protein) [16], *PUMA* (p53-upregulated modulator of apoptosis) [17], and *BID* (BH3-interacting domain) [18]. However, p53-induced apoptosis is more complex, involving the transcriptional activation of multiple pro-apoptotic molecules that execute apoptotic functions in a tissue-cell-specific and context-dependent manner [19,20]. Thus, discovering and characterizing novel p53 targets involved in DNA damage-induced apoptosis will enhance our understanding of the p53-mediated apoptotic program in response to DNA damage in different cellular contexts.

NEMF is an essential component of the ribosome quality control complex (RQC) [8]. Within the RQC complex, the NEMF specifically binds to stalled 60S ribosomal subunits and facilitates the recognition and ubiquitination of stalled 60S subunits by the Listerin E3 Ubiquitin Protein Ligase 1 (LTN1) [21]. Dysregulation of NEMF has been implicated in various cancers, including colon cancer and ovarian cancer [22]. In addition to cancer, NEMF has also been linked to neuromuscular diseases and contributes to the maintenance of cellular homeostasis and the coordination of cellular response to oxidative stress, RNA damage and DNA damage [23–25]. *Drosophila caliban* (*clbn*) is the ortholog of the human *NEMF* [22]. Initially identified as a nuclear exporting mediator for Prospero in S2 cells, Clbn also functions as a tumor suppressor in human non-small cell lung cancer (NSCLC) cells [26]. Our previous studies demonstrated that *clbn*-deficient flies are highly sensitive to DNA damage induced by ionizing radiation. Transcription of *clbn* gene is activated after irradiation and Clbn is found to play important roles in regulating both p53-dependent and -independent apoptosis induced by DNA damage [27]. Moreover, Clbn mediates the G1/S checkpoint by repressing E2F1 activity [28]. Recent studies have also identified Clbn as a component of the RQC complex that mediates CAT tailing by recruiting alanine-charged tRNA [29]. Clbn also regulates mitochondrial dynamics in enterocytes (ECs) and is required for the maintenance of intestinal homeostasis [30]. Despite the diverse roles of NEMF/Clbn in regulating multiple biological processes, how its expression is regulated after DNA damage remains largely unknown. Clbn reveals functional conservation with human ortholog NEMF, suggesting that *Drosophila* can serve as a model organism to provide insights into the physiological function and transcriptional regulation of NEMF. In this study, we investigated the mechanism of *clbn* transcriptional regulation in response to DNA damage and provided direct evidences that *clbn* is a direct target transcriptionally activated by p53 in response to DNA damage.

## Results

### Characterization of the critical promoter region of the *clbn* gene

**Fig 1:**
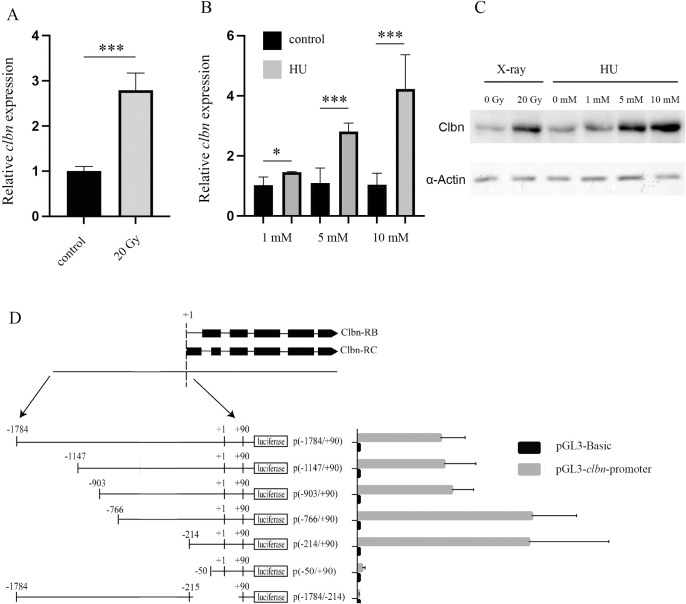
Identification of the critical promoter region of the *clbn* gene. *clbn* mRNA level was analyzed by qRT-PCR. The housekeeping gene *β-tubulin* was used as an internal control for normalization. Clbn expression was upregulated following treatment with 20 Gy X-ray (A) or HU (B). qRT-PCR data were shown as mean ± SEM for three independent experiments. *P < 0.05, ***P < 0.001. (C) Clbn protein level was analyzed by western-blot. Clbn expression was upregulated following treatment with 20 Gy X-ray or HU. (D) Characterization of the *clbn* promoter region by dual luciferase reporter assays. The numbers indicate the positions relative to the transcription start site of *clbn* gene. The transcriptional activity of each construct was shown as the luciferase activity relative to that of the pGL3-Basic vector. Values were normalized to the Renilla activity and were given as means ± SEM for three independent experiments.

Our previous study demonstrated that the expression of *clbn* is enhanced following DNA damage induced by ^60^Co γ-rays irradiation [27]. To further confirm that *clbn* expression is robustly induced by DNA damage, we treated *Drosophila* S2 cells with X-ray irradiation and Hydroxyurea (HU), two additional widely used DNA-damaging agents. As shown in Fig 1A, X-ray treatment (20 Gy) significantly increased *clbn* mRNA expression in S2 cells. Similarly, HU treatment, which induces replicative stress and DNA damage, enhanced *clbn* expression in a dose-dependent manner (Fig 1B). The expression of *mre11* gene, a well characterized DNA damage response gene, was upregulated by X-ray and HU treatment and served as a positive control (S1 Fig). Consistent with the increased *clbn* expression at mRNA level following X-ray or HU treatment, the Clbn protein level was also upregulated in response to DNA damage (Fig 1C). These results, combined with our previous findings^27^, strongly suggest that *clbn* is a DNA damage-responsive gene.

To explore the molecular basis of *clbn* mRNA induction by DNA damage, the promoter region of *clbn* was characterized using luciferase reporter assay. A 1875-bp fragment spanning the 5′ flanking region of *clbn* gene was cloned into the luciferase reporter plasmid. This plasmid was then transfected into *Drosophila* S2 cells, and the promoter activity was examined. As shown in Fig 1C, the cloned fragment showed significantly higher luciferase activity than that of the control construct, indicating that an active promoter region of the *clbn* gene was isolated. To precisely map the minimal active promoter, a series of deletion constructs were constructed. Removal of the 5′ end of (−1784/ + 90) to −50 or the 3′ end of (−1784/ + 90) to −215 completely abolished reporter gene activity, localizing the minimal promoter of *clbn* gene between −214 and +90 relative to the transcription start site (Fig 1D).

### *clbn* promoter activity is upregulated in response to DNA damage

**Fig 2:**
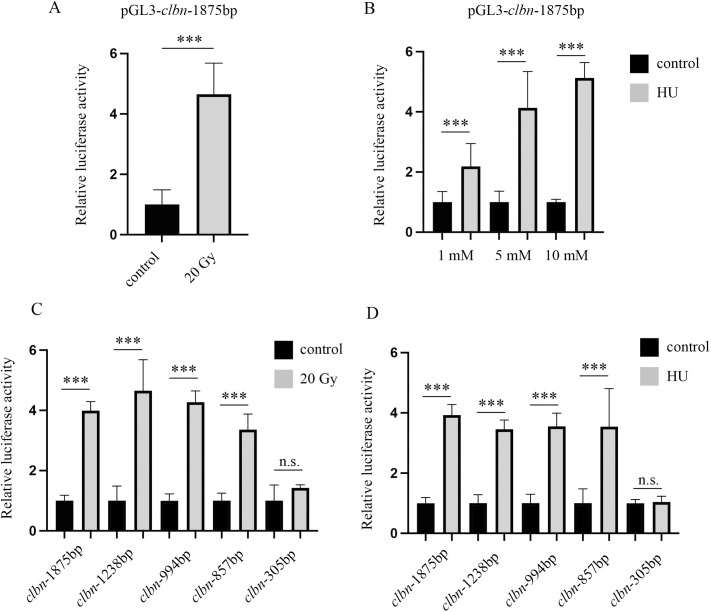
*clbn* promoter activity is promoted following DNA damage. The full-length *clbn* promoter activity was induced by treatment with 20 Gy X-ray (A) or HU (B). The activity of series of deletion constructs following treatment with 20 Gy X-ray (C) or 10mM HU (D) was analyzed. Deletion of the 5′ region from −766 to −215 completely abolished promoter activity induction by DNA damage. The data were shown as means ± SEM for three independent experiments. ***P < 0.001. n.s., not significant.

To investigate the mechanisms underlying induced *clbn* expression following DNA damage, the full-length *clbn* promoter construct was transfected into *Drosophila* S2 cells, and the promoter activity was assessed following DNA damage induced by X-ray or HU. As shown in Fig 2A, *clbn* promoter activity was significantly elevated in response to X-ray-induced DNA damage. Similar results were obtained when treating S2 cells with HU (Fig 2B), indicating that the *clbn* promoter is activated by DNA damage. To identify the region responsible for DNA damage-induced upregulation of *clbn* promoter activity, several *clbn* promoter truncation constructs were transfected into S2 cells followed by X-ray or HU treatments. As shown in Fig 2C and 2D, deletion of the 5′ region from −766 to −215 completely abolished promoter activity induction by DNA damage, although this deletion did not affect promoter activity under normal conditions. These results demonstrate that the *clbn* promoter is activated by DNA damage and that the promoter region from −766 to −215 is essential for *clbn* promoter activation during DNA damage response.

### *clbn* expression is positively regulated by p53

**Fig 3:**
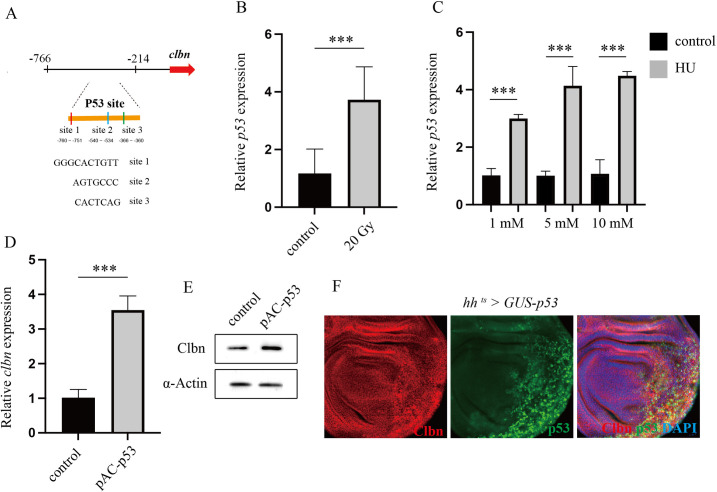
Clbn expression is positively regulated by p53. (A) Schematic diagram of three putative p53-binding sites (site 1, site 2 and site 3) within the *clbn* promoter. *p53* mRNA level was analyzed by qRT-PCR. The housekeeping gene *β-tubulin* was used as an internal control for normalization. The *p53* expression was elevated following treatment with 20 Gy X-ray (B) or HU (C). The S2 cells were transiently transfected with pAC-p53. The *clbn* mRNA level and Clbn protein level were analyzed by qRT-PCR (D) and western-blot (E) respectively. Wing disc showing expression of Clbn in the posterior region with *p53* over-expression driven by *hh* ^*ts*^ > Gal4. Clbn staining is shown in red, and p53 expression is shown in green. Expression of Clbn is upregulated by *p53* over-expression (F). The data were shown as means ± SEM for three independent experiments. ***P < 0.001.

To identify the key regulators of the *clbn* promoter activity in response to DNA damage, the promoter sequence of *clbn* from −766 to −215 was analyzed using the Promo analytical tool. As shown in Fig 3A, this region contains three putative p53-binding sites. Given that p53 is a central transcriptional regulator of genes involved in the DNA damage response, we investigated whether p53 regulates the transcription of *clbn*. Consistent with previous findings, the expression of *p53* was increased by X-ray or HU-induced DNA damage (Figs 3B and 3C). As shown in Fig 3D and 3E, both of *clbn* mRNA and protein levels are elevated following the over-expression of *p53*. Meanwhile, induced expression of *p53* in the posterior compartment of wing imaginal disc using *hh-Gal4*^*ts*^ resulted in a significant upregulation of Clbn protein level (Fig 3F). Furthermore, the expression of the human *NEMF* was also upregulated by overexpression of *TP53* (S2 Fig), indicating a conserved regulation of *NEMF/clbn* expression by p53 between human and *Drosophila*. Together, these results indicate that p53 positively regulates the expression of *clbn* at both mRNA and protein level.

### p53 protein directly binds and transactivates the *clbn* promoter

**Fig 4:**
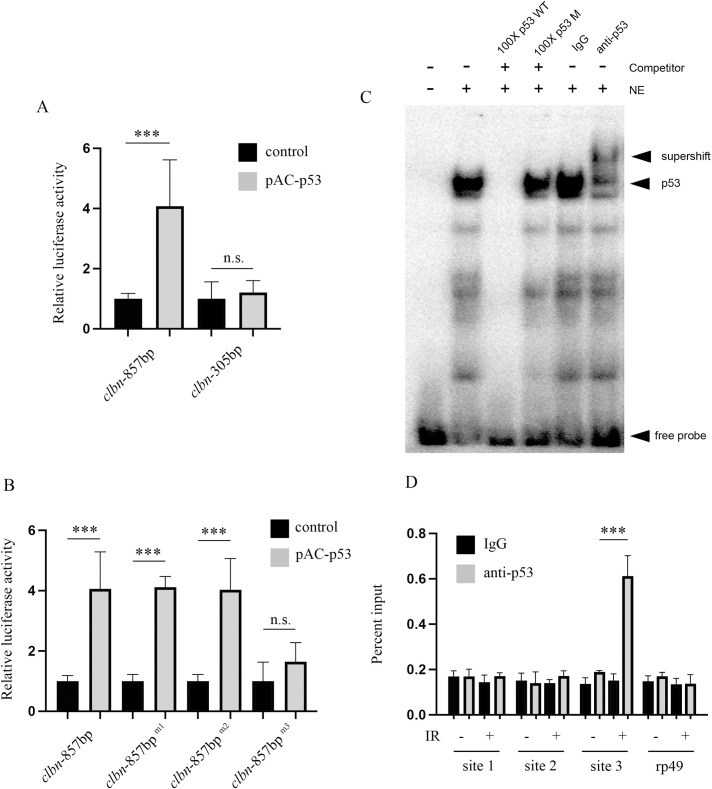
p53 directly binds to *clbn* promoter. (A) Overexpression of *p53* upregulates *clbn* promoter activity. (B) The S2 cells were transiently transfected with pAC-p53 and promoter construct with p53-binding site mutated individually (m1, m2 and m3). The activity of the construct was analyzed by dual luciferase assays. The activity of each construct was shown as the luciferase activity relative to that of promoter construct co-transfected with the control vector. The data were shown as means ± SEM for three independent experiments. ***P < 0.001. (C) Electrophoretic mobility shift assay (EMSA) analysis of p53 binding to site 3 within *clbn* promoter *in vitro*. Biotin-labeled DNA probes were incubated with the nuclear extracts (NE) from S2 cells treated with X-ray. Unlabeled DNA probes (100 × molar excess) of the wild-type (p53 WT) or with the mutated site 3 (p53 M) were added as competitors (lanes 3 and 4). The p53 antibody was added to supershift p53-DNA complexes (lanes 6). (D) ChIP assay was performed using S2 cells treated with 20 Gy X-ray. DNA-protein complexes were immunoprecipitated with p53 antibody or control IgG, and immunoprecipitation of p53 associated DNA fragments were analyzed by qPCR with primers flanking three p53-binding sites respectively. The data were shown as means ± SEM for three independent experiments. ***P < 0.001.

The *clbn* promoter harbored three putative p53-binding sites, and *clbn* expression was positively regulated by p53. We therefore examined whether p53 can activate the *clbn* promoter activity. As shown in Fig 4A, over-expression of *p53* in S2 cells significantly elevated *clbn* promoter (−766/ + 90) activity by approximately 4-fold. Deletion of the −766 to −215 region attenuated promoter activation by *p53* overexpression, further suggesting the critical role of this region in p53-mediated activation of the *clbn* promoter. To further characterize the function of these three putative p53 binding sites in *clbn* promoter, we individually mutated each of the three p53 binding sites in the reporter construct (−766/ + 90). These constructs were transfected into S2 cells and the promoter activity were examined. As shown in Fig 4B, mutation of p53-binding site 1 or 2 had no significant effect on promoter activity induced by *p53* overexpression. However, mutation of p53-binding site 3 almost completely abolished p53-mediated induction of *clbn* promoter activity. These results indicate that the p53-binding site located at −366/360 is essential for p53 activation of the *clbn* promoter.

We next measured whether p53 can directly bind to the p53-binding site 3 within the *clbn* promoter. We performed electrophoretic mobility shift assays (EMSA) using nuclear extract from X-ray treated S2 cells and biotin-labeled double-stranded DNA probes covering the site 3. Incubation of the wildtype probe with the nuclear extract generated a distinct EMSA signal, indicating protein-DNA complex formation. This signal was effectively competed by the addition of excess unlabeled wildtype probe, confirming binding specificity. In contrast, the signal persisted when using a competitor probe harboring a mutation in the site 3, demonstrating that the observed interaction is dependent on an intact p53 consensus sequence. Moreover, the addition of anti-p53 antibody induced a supershift of the protein-DNA complex, providing direct evidence that p53 is a constituent of the detected protein-DNA complex (Fig 4C). These results collectively demonstrate that site 3 harbors a functional p53 response element capable of recruiting p53 *in vitro*. We next performed the chromatin immunoprecipitation (ChIP) assay to characterize the interaction between p53 and the *clbn* promoter *in vivo*. As shown in Fig 4D, under unstressed condition, p53 only weakly binds to the site 3 within *clbn* promoter region. However, after DNA damage, significant binding of p53 to site 3 within *clbn* promoter was observed, with no binding detected at site 1 or site 2. These data demonstrated that *clbn* gene is a direct transcriptional target of p53 in response to DNA damage.

### p53 is required for enhanced expression of Clbn after DNA damage

**Fig 5:**
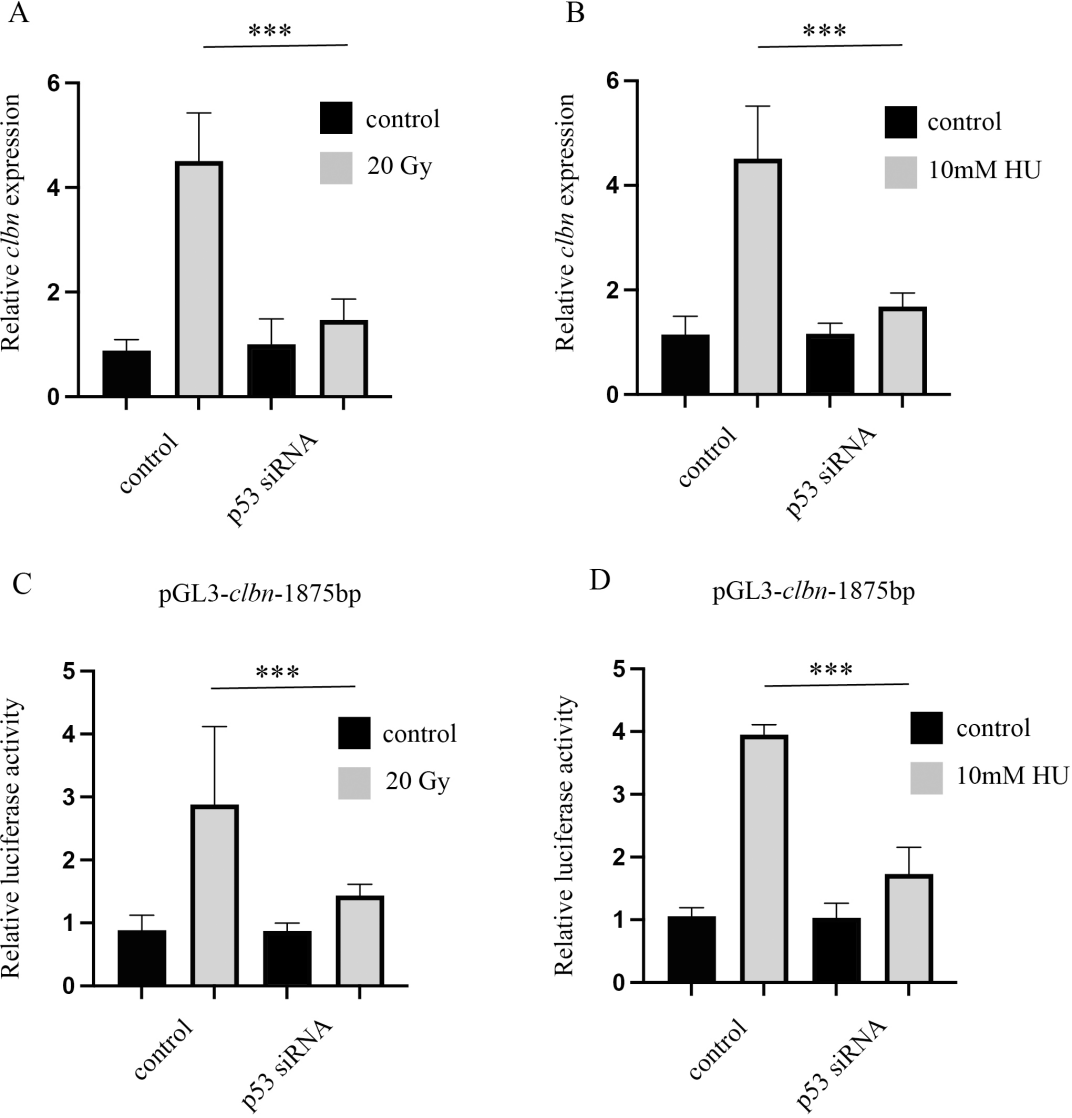
p53 is required for Clbn induction in response to DNA damage. S2 cells were transfected with p53 siRNA or control siRNA. The DNA damage was induced by treatment with X-ray (A) or HU (B). *clbn* mRNA level was analyzed by qRT-PCR. The housekeeping gene *β-tubulin* was used as an internal control for normalization. S2 cells were co-transfected with the *clbn* promoter construct and p53 siRNA or control siRNA. The DNA damage was induced by X-ray (C) or HU (D). The promoter activity of the construct was analyzed by dual luciferase assays. The data were shown as means ± SEM for three independent experiments. ***P < 0.001.

We further explored whether p53 is required for *clbn* upregulation in response to DNA damage. As illustrated in Fig 5A, *clbn* mRNA induction triggered by DNA damage was impaired by knockdown of *p53* expression (Figs 5A, 5B and S3 Fig). Similarly, DNA damage-induced activation of the *clbn* promoter was diminished when *p53* expression was knocked down (Figs 5C and 5D). These results suggested that upregulation of *clbn* following DNA damage is p53-dependent, and Clbn may function as part of the DNA damage response network mediated by p53.

## Discussion

The p53 tumor suppressor acts as a critical orchestrator of cellular responses to DNA damage. However, the full repertoire of transcriptional targets that dictate p53-mediated DNA damage responses remains incompletely characterized [31]. Mutations in p53 are the most prevalent genetic alterations in human cancers, with the majority mapping to its DNA-binding domain, underscoring the pivotal role of p53’s transcriptional activity in tumor suppression [32,33]. A central aim of p53 research has been to delineate the specific target genes that govern cell cycle arrest and apoptosis, two cardinal processes in the DNA damage response. Model organisms such as *Drosophila* continue to offer valuable insights into conserved mechanisms of DNA damage sensing and repair, given the evolutionary conservation of p53-dependent gene regulation. In this study, we uncover novel aspects of the transcriptional regulation of *clbn* in response to DNA damage. First, we characterized the *clbn* promoter and demonstrated that *clbn* expression and promoter activity are induced by DNA damage. Second, we identified a canonical p53 response element within the *clbn* cis-regulatory region, showing that p53 directly binds this site to activate the transcription of *clbn*. Third, overexpression of human p53 upregulated *NEMF*, the human ortholog of *clbn*, indicating a conserved regulatory axis between p53 and *NEMF*/*clbn* across species. These findings establish *clbn* as a novel transcriptional target of p53 and a key component of the p53-mediated DNA damage response network.

The *NEMF/clbn* encodes a component of the RQC complex, which resolves ribosome collisions to mitigate their cytotoxic effects. Emerging studies indicate that exposure to DNA-damaging agents induces ribosome stalling, thereby activating the RQC pathway [34,35]. The RQC and DNA damage response (DDR) pathways are deeply interconnected, with reciprocal regulatory influences at multiple levels; their dysregulation is linked to diverse pathologies, including cancer and neurodegenerative diseases [36]. The *NEMF* expression exhibits tissue-specific variability across cancer types: it is downregulated in most malignancies (e.g., brain, central nervous system, breast, colon, and stomach cancers) but shows distinct patterns in other contexts. Mechanistic studies suggest NEMF modulates cancer progression through pathways like PI3K/MTOR signaling, for instance, in ovarian cancer, *NEMF* overexpression inhibits cell proliferation and migration by suppressing this pathway, inducing cell cycle arrest and apoptosis, implying a potential tumor-suppressive role [25]. Our prior work identified *Drosophila* Clbn as a tumor suppressor, integrating into a regulatory network with p53, caspases, and Hid proteins during DNA damage-induced apoptosis [27]. The current study reveals that human TP53 upregulates *NEMF* expression; bioinformatic analysis of the *NEMF* promoter region identified putative p53-binding sites (S4 Fig), and data from the Harmonizome database (https://maayanlab.cloud/Harmonizome/) further support *NEMF* as a transcriptional target of TP53. These findings suggest conservation not only in *clbn* function between *Drosophila* and humans but also in p53-mediated regulation of *NEMF/clbn*. Future research should validate p53-dependent transcriptional control of human *NEMF* across diverse cell types and stress conditions.

In summary, our present study established *clbn* as a p53-responsive gene participating in the p53-dependent transcriptional program in response to DNA damage, providing new insights into the interplay between RQC and DDR pathways. Further research is needed to fully elucidate the molecular mechanisms underlying *p53*-*NEMF*/*clbn* regulatory axis, as well as its dysregulation in various diseases. This knowledge may pave the way for the development of novel therapeutic strategies for treating diseases where the RQC-DDR axis is disrupted.

## Materials and methods

### Fly genetics

All flies were reared at 25 °C and 65% humidity on standard corn meal unless specified. The following fly lines were used in this study: *hh-Gal4, Gal80*^*ts*^*; GUS-p53*. All fly lines were obtained from Bloomington Stock Center*.*

### Cells culture and treatment

*Drosophila* S2 cells were grown at 25 °C in Schneider’s *D*rosophila medium (Thermo Fisher) supplemented with 10% (v/v) heat-inactivated FBS (Gibco), 50 units/mL penicillin, and 50ug/mL streptomycin. To induce DNA damage, log phase S2 cells were exposed to X-ray radiation at a dose of 20 Gy using (Dandong Aolong Radiative Instrument Co., Ltd, China) or treated with HU (Sigma-Aldrich) at different concentrations (1mM, 5mM and 10mM), which can produce many types of DNA lesions, such as single-strand breaks (SSBs) and double-strand breaks (DSBs). HeLa cells are routinely cultured in Dulbecco’s Modified Eagle Medium (DMEM) (Sigma, D6546) high Glucose supplemented with 10% FBS at 37 °C and 5% CO2.

### Plasmid construction

The genomic fragment (−1784 to +90, relative to the transcription start site of *clbn* gene) of *clbn* gene was amplified by PCR using LA Taq polymerase (Takara) and the genomic DNA isolated from S2 cells as the template. The fragment was then cloned into the pGL3-Basic vector (Promega). A series of 5’ or 3’ deletions of the *clbn* promoter region were generated by PCR using the construct (−1784 to +90) as the template, and the sequences of the constructs were verified via Sanger sequencing. Primers for plasmid construction are listed in S1 Table. Site-directed mutagenesis of p53 binding site (site1, site2 or site3) in *clbn* promoter construct (−766 to +90) was performed using GeneArt Site-Directed Mutagenesis System (Thermo Fisher) following manufacturer’s guidelines, and the mutagenesis was sequence-verified via Sanger sequencing.

### Transfections and luciferase reporter assays

For transient transfection of S2 cells, cells were seeded at a density of 6 × 10^5^ cells/well in a 24-well plate. On the following day, the cells were transfected with 0.2 μg of luciferase reporter plasmid using Effectene Transfection Reagent (QIAGEN) following manufacturer’s guidelines. The pAC5.1-Renilla plasmid was co-transfected with the luciferase reporter plasmid, and used as an internal control for luciferase activity normalization. For p53 overexpression, 0.3 ug of pAC-p53 or control plasmids were co-transfected with the luciferase reporter plasmids. For p53 knockdown, 50 nM of p53 or control siRNA were co-transfected with the luciferase reporter plasmids. The cells were treated with X-ray or HU 48 hours after the transfection. For luciferase reporter assays, the cell lysates were prepared and luciferase activity was measured with the Dual-Luciferase Reporter Assay system (Promega) using the luminometer (Bio-rad) according to the manufacturer’s instructions. For transient transfection of Hela cells, cells were seeded at a density of 2 × 10^5^ cells/well in a 6-well plate. On the following day, the cells were transfected with 0.5 μg of pCMV-p53 or control plasmids using Effectene Transfection Reagent (QIAGEN) following manufacturer’s guidelines. Primers and siRNAs sequences were listed in S1 Table. All experiments were performed in triplicate wells and repeated three times.

### Western-blot

Protein samples were separated by SDS-PAGE and transferred onto polyvinylidene fluoride membranes. The membranes were immunoblotted with rabbit anti-Clbn antibody (1:2,000) [30] and mouse anti-α-actin antibody (1:2,000; JLA20, Developmental Studies Hybridoma Bank) individually. Following this, the blots were incubated with horseradish peroxidase-conjugated goat anti-rabbit or goat anti-mouse secondary antibodies (GE Healthcare), and chemiluminescence detection was performed using GE Healthcare reagents.

### Electrophoretic mobility shift assay

Nuclear extracts were isolated from S2 cells treated with 20 Gy X-ray following the method outlined in a prior study [37]. To generate the wild-type DNA probe targeting the *clbn* promoter (spanning positions −381 to −340), two complementary oligonucleotides were annealed. For the mutated probe of the *clbn* promoter, the same annealing procedure was employed, with the exception of introducing a mutation at site 3. Oligo sequences were listed in S1 Table. A 10 μg aliquot of nuclear extracts was preincubated in 20 μl of binding buffer (10 mM Tris-HCl (pH 7.5), 50 mM NaCl, 1 mM MgCl2, 0.5 mM EDTA, 0.5 mM dithiothreitol, 50 μg/ml poly(dI-dC)·poly(dI-dC), and 4% glycerol). This preincubation was conducted either with or without an unlabeled competitor (at a 100-fold molar excess). In supershift assays, 1 μg of p53 antibody or IgG (Sigma) was included in the preincubation mixture. After 30 minutes of preincubation on ice, the biotin-labeled DNA probe was added, and the samples were incubated at room temperature for 30 minutes. Subsequently, the reaction mixtures were separated using 4% polyacrylamide gels and transferred to a nylon membrane. The labeled oligonucleotides were detected with the LightShift Chemiluminescent EMSA Kit (Thermo Fisher) following the instructions of the manufacturer.

### Chromatin immunoprecipitation assay

ChIP assay was performed as previously described [38]. Briefly, S2 cells were cross-linked with formaldehyde at a final concentration of 1% and incubated for 10 min at room temperature. After quenching the reaction, the cells were harvested, and resuspended in lysis buffer (1% SDS/10 mM EDTA/50 mM Tris–HCl, pH 8.1). The cell lysate was sonicated to shear DNA to an average fragment size of 200−1000 bp, and the supernatant was collected after centrifugation. Dilute the chromatin 10-fold with IP dilution buffer (0.1% Triton X-100, 20 mM tris- HCl (pH 8.0), 2 mM EDTA, 150 mM NaCl) supplemented with protease inhibitors. After an overnight incubation with the Dmp53 antibody (generated by our lab) at 4 °C, the immunocomplexes were captured using Protein A Dynabeads (Invitrogen) and then washed four times with washing buffer [20 mM tris- HCl (pH 8.0), 250 mM NaCl, 1 mM EDTA, and 0.5% NP-40] and once with Tris-EDTA (TE) buffer. The chromatin complex was eluted from the beads with 200 μl of freshly prepared elution buffer (1% SDS, 0.1 M NaHCO3). After elution, the samples were reverse cross-linked by incubation at 65°C for 6 hours. The chromatin DNA was purified with the Qiaquick PCR purification kit (Qiagen) and resuspended in elution buffer. Input and eluate DNA were subjected to quantitative real-time PCR. Primers used for qPCR were listed in S1 Table.

### Immunostaining and imaging

The wing imaginal disc from 3^rd^ larva was dissected in cold PBS and fixed immediately 4% formaldehyde freshly prepared in PBS. Samples were then washed three times in PBT buffer (PBS with 0.1% Triton X-100) and blocked in PBT supplemented with 5% (vol/vol) normal goat serum. The samples were incubated with primary antibodies overnight at 4 °C. The primary antibodies were used as follows: rabbit anti-Clbn (1:400) [30], and guinea pig anti-Dmp53 (1:200) (Abgent Biotechnology, Suzhou, China). Following primary antibody incubation, the samples were washed three times with PBT buffer, and then incubated with secondary anti-rabbit Alexa 555 or anti-guinea pig Alexa 488 fluorescence antibodies (1:400, Cell Signaling Technology). Samples were washed four times with PBT buffer and mounted on a slide. The pictures were captured using an Olympus FV1000 confocal laser-scanning microscope. The images were processed using Adobe Photoshop, ImageJ, and Illustrator.

### RT-qPCR

Total RNA of cells was isolated using TRIzol reagent (Invitrogen), and purifed with RNAeasy kit (Qiagen). Following RNA extraction, the complementary DNA (cDNA) was synthesized using the iScript cDNA synthesis kit (Bio-Rad). Quantitative PCR was performed with the iScript one step RT-PCR SYBR green kit (Bio-Rad). The β-tubulin gene was used as the internal control for normalization. Three independent biological replicates of the experiment were conducted. All results were presented as mean ± SEM. Primers used for RT-qPCR were listed in S1 Table.

### Statistics

All statistical comparisons were performed using Prism 9.0. Unpaired two-tailed Student’s t-test was used to assess statistical significance between two conditions. Raw data were included in S2 Table. The significance level was indicated as ^*^ for **P* *< 0.05, and ^***^ for **P* *< 0.001.

## Supporting information

S1 Fig The *mre11* expression was upregulated following DNA damage. Treatment with 20 Gy X-ray (A) or HU (B) induced *mre11* expression. The housekeeping gene *β-tubulin* was used as an internal control for normalization. qRT-PCR data were shown as mean ± SEM for three independent experiments. ***P < 0.001.
(DOCX)

Table S1 Primers and oligos used in this study. (XLSX)

Table S2 Raw data. (XLSX)
