# Supplementary material for: Caliban is a transcriptional target of p53 in response to DNA damage
Source: PLoS One. 2025 Aug 28;20(8):e0331141. doi: 10.1371/journal.pone.0331141 (PMC12393737; doi:10.1371/journal.pone.0331141)
Supplement: S1 Fig — Treatment with 20 Gy X-ray (A) or HU (B) induced mre11 expression. The housekeeping gene β-tubulin was used as an internal control for normalization. qRT-PCR data were shown as mean ± SEM for three independent experiments. ***P < 0.001. (DOCX) [file pone.0331141.s001.docx]

**Supplementary figures**


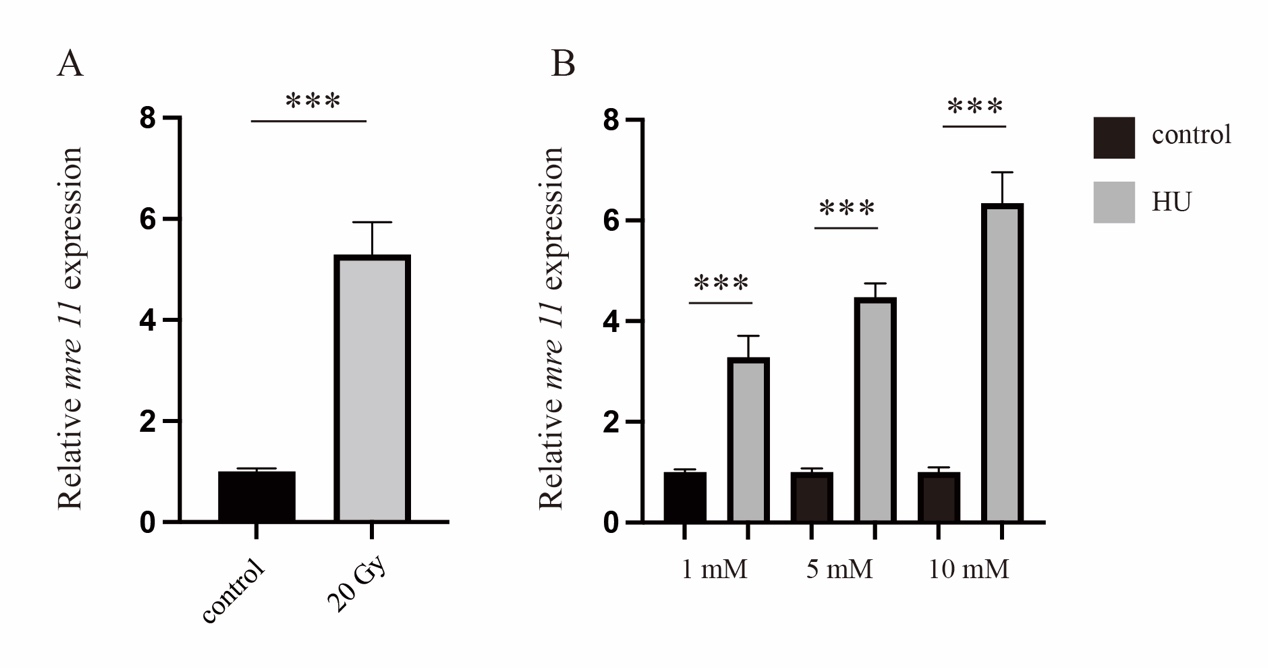


**Figure S1. The *mre11* expression was upregulated following DNA damage.** Treatment with 20 Gy X-ray (A) or HU (B) induced *mre11* expression. The housekeeping gene *β-tubulin* was used as an internal control for normalization. qRT-PCR data were shown as mean ± SEM for three independent experiments. ****P* < 0.001.

**
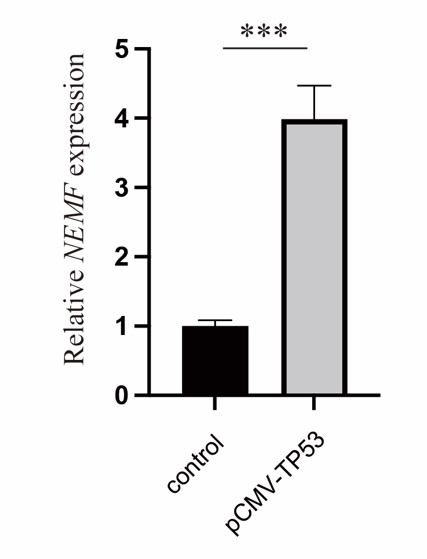
**

**Figure S2. The human *NEMF* expression is enhanced by overexpression of human TP53.** Hela cells were transfected with pCMV-TP53 or control vector. *NEMF*mRNA level was analyzed by qRT-PCR. The housekeeping gene *β-tubulin* was used as an internal control for normalization. The data were shown as means ± SEM for three independent experiments. ****P* < 0.001.

**
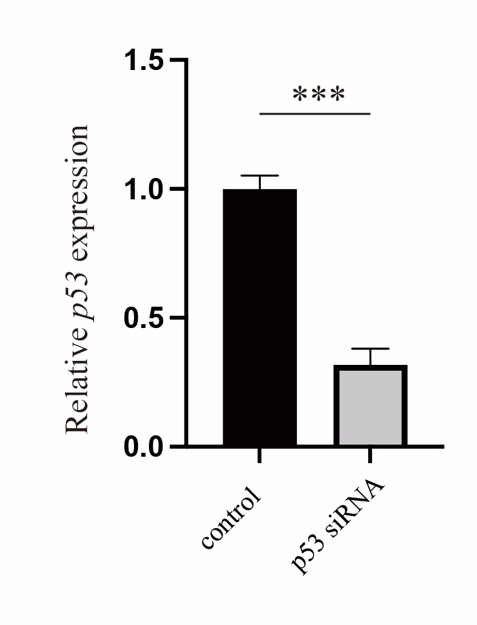
**

**Figure S3. Knockdown of *p53* expression.** S2 cells were transfected with p53 siRNA or control siRNA. *p53*mRNA level was analyzed by qRT-PCR. The housekeeping gene *β-tubulin* was used as an internal control for normalization. The data were shown as means ± SEM for three independent experiments. ****P* < 0.001.


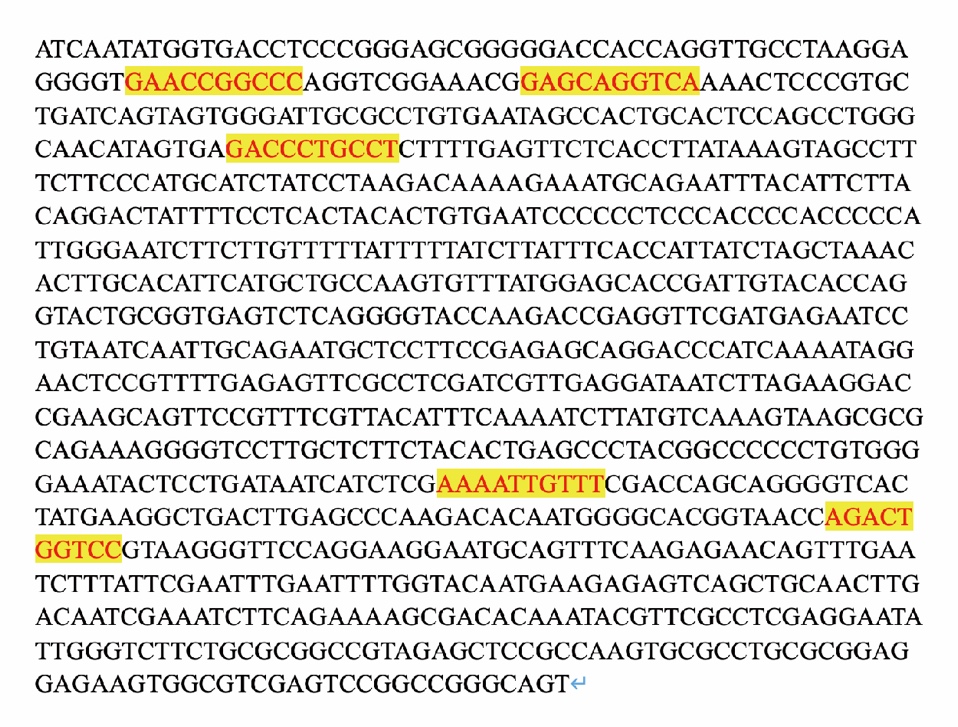


**Figure S4. Analysis of putative p53-binding sites within 1 kb upstream of the human *NEMF* gene.** The putative p53-binding sites are identified by bioinformatic analysis (TFBIND, https://tfbind.hgc.jp/) and highlighted in yellow.
